# The validation and utility of a quantitative one-step multiplex RT real-time PCR targeting Rotavirus A and Norovirus

**DOI:** 10.1016/j.jviromet.2012.09.021

**Published:** 2013-01

**Authors:** Tran Thi Ngoc Dung, Voong Vinh Phat, Tran Vu Thieu Nga, Phan Vu Tra My, Pham Thanh Duy, James I. Campbell, Cao Thu Thuy, Nguyen Van Minh Hoang, Pham Van Minh, Hoang Le Phuc, Pham Thi Ngoc Tuyet, Ha Vinh, Duong Thi Hue Kien, Huynh Le Anh Huy, Nguyen Thanh Vinh, Tran Thi Thu Nga, Nguyen Thi Thu Hau, Nguyen Tran Chinh, Tang Chi Thuong, Ha Manh Tuan, Cameron Simmons, Jeremy J. Farrar, Stephen Baker

**Affiliations:** a The Hospital for Tropical Diseases, Wellcome Trust Major Overseas Programme, Oxford University Clinical Research Unit, Ho Chi Minh City, Viet Nam; b Centre for Tropical Diseases, University of Oxford, Oxford, United Kingdom; c Children's Hospital 1, Ho Chi Minh City, Viet Nam; d Children's Hospital 2, Ho Chi Minh City, Viet Nam; e The Hospital for Tropical Diseases, Chi Minh City, Viet Nam

**Keywords:** Rotavirus, Norovirus, Real-time PCR, Quantitation, Internally controlled

## Abstract

Rotavirus (RoV) and Norovirus (NoV) are the main causes of viral gastroenteritis. Currently, there is no validated multiplex real-time PCR that can detect and quantify RoV and NoV simultaneously. The aim of the study was to develop, validate, and internally control a multiplex one-step RT real-time PCR to detect and quantify RoV and NoV in stool samples. PCR sensitivity was assessed by comparing amplification against the current gold standard, enzyme immunoassay (EIA), on stool samples from 94 individuals with diarrhea and 94 individuals without diarrhea. PCR detected 10% more RoV positive samples than EIA in stools samples from patients with diarrhea. PCR detected 23% more NoV genogroup II positive samples from individuals with diarrhea and 9% more from individuals without diarrhea than EIA, respectively. Genotyping of the PCR positive/EIA negative samples suggested the higher rate of PCR positivity, in comparison to EIA, was due to increased sensitivity, rather than nonspecific hybridization. Quantitation demonstrated that the viral loads of RoV and NoV in the stools of diarrheal patients were an order of magnitude greater than in individuals without diarrhea. This internally controlled real-time PCR method is robust, exhibits a high degree of reproducibility, and may have a greater utility and sensitivity than commercial EIA kits.

## Highlights

► A multiplex real-time PCR was developed for the detection and quantitation of Rotavirus A and Norovirus genogroup II. ► An internal extraction and amplification control was incorporated. ► Real-time PCR was compared to the current gold standard, enzyme immunoassay. ► Real-time PCR was significantly more sensitive than enzyme immunoassay. ► Quantitation demonstrated that the viral loads of both pathogens were ten times greater in stools children with diarrhea than in children without diarrhea.

## 1 Introduction

Diarrhea remains a major cause of childhood morbidity and mortality globally (Thapar et al., 2004; Wardlaw et al., 2010), with the vast majority of the 2 billion annual infections and 2.5 million resulting deaths occurring in low and middle-income countries. Viruses, specifically Rotavirus A (RoV) and genogroup I and II Norovirus (NoVI and II) are predominant causes of viral gastroenteritis worldwide, and are responsible for over 40% of all cases of diarrhea in developing countries (Davidson et al., 2002; Patel et al., 2008; Widdowson et al., 2009). A variety of techniques are used to detect RoV and NoV in stool samples, including electron microscopy (Caul, 1996a,b), latex agglutination (Lee et al., 2010), PCR amplification, and enzyme immunoassay (EIA) (Jiang et al., 2000). Commercial EIA kits are the most common methods used for diagnosis, as they offer simplicity and good specificity, but may lack overall sensitivity in some settings (Barreira et al., 2010; Ramani et al., 2010). Typically, the sensitivity of viral detection in biological material can be increased using molecular methods (Freeman et al., 2008; Kageyama et al., 2003), with real-time reverse transcriptase (RT) PCR having advantages over conventional RT-PCR (Freeman et al., 2008; Trujillo et al., 2006). The aim of the study was to develop and validate a multiplex one-step RT real-time PCR, controlled internally, to detect and quantify RoV and NoV in stool samples from individuals with and without diarrhea.

## 2 Materials and methods

### 2.1 Ethics statement

This study was conducted according to the principles expressed in the Declaration of Helsinki, and was approved by the ethical review boards of the Hospital for Tropical Diseases (HTD), Children's Hospital 1 (CH1) and Children's Hospital 2 (CH2) in Ho Chi Minh City in Viet Nam, and the Oxford Tropical Research Ethics Committee (OxTREC) in the United Kingdom. An informed consent form, signed by a parent or guardian, was required for participation.

### 2.2 Stool samples and sample processing

Stool samples were obtained from a group of symptomatic children (with diarrhea) and a group of asymptomatic children (without diarrhea). The symptomatic group consisted of 217 children, aged between 0 and 60 months, admitted to CH1, CH2 or HTD between May 2009 and April 2010 with acute watery diarrhea (defined as three or more loose stools without blood within a 24 h period). The asymptomatic group consisted of 277 children, aged between 0 and 60, attending CH2 for health checks between May 2009 and April 2010 without diarrhea. Stool specimens were collected from all participants within 24 h of hospital admission and prior to antimicrobial treatment. All samples were stored at 4 °C before transportation to the laboratory on the same day as collection. Samples were assayed to detect RoV and NoV using ProspecT Rotavirus and IDEIA Norovirus EIA tests following manufacturer's recommendations (Oxoid, United Kingdom). Two aliquots of each sample were stored at −80 °C as 10% suspensions in distilled phosphate buffered saline (PBS).

### 2.3 Equine arterititis virus (EAV)

EAV was added to all samples prior to nucleic acid extraction as an internal control (Hue et al., 2011). Aliquots of post culturing supernatant containing EAV were prepared as described previously (Hue et al., 2011; Scheltinga et al., 2005). The precise amount of virus added to the stool samples was assessed by PCR titration. The final EAV dilution used in the PCR assay produced *Cp* values between 30 and 33. Amplification runs that did not produce a *Cp* value within this range for EAV were discarded and samples were subjected to a secondary extraction and amplification.

### 2.4 NoV and RoV PCR amplification primers and probes

Primers, probes and their optimal concentrations are described in Table 1. Briefly, the RoV primers targeted the gene encoding non-structural protein 3 (NSP3) (Freeman et al., 2008), and the probe incorporated a FAM reporter and a BHQ1 quencher. The NoV primers and probes targeted the ORF1-ORF2 junction of NoV I and NoVII (Trujillo et al., 2006). The reporter of the NoVII probe was adapted from the probe published in Trujillo et al. (2006) from Quasar 670 (red) to Cyan 500 (blue–green) as these probes have a higher performance using the LightCycler format and as a consequence of the Cy5 tag of the internal control. The primers and probe for EAV were as previously described (Hue et al., 2011; Scheltinga et al., 2005).

### 2.5 Nucleic acid extractions and one-step RT Real-time PCR

Nucleic acid was extracted from bacterial and viral sources using the Wizard Nucleic Acid Purification Kit (Promega, USA) and the QIAamp Viral RNA Mini Kit (QIAGEN, USA), respectively. Nucleic acid preparations were diluted to a concentration of 20 ng/μl, and stored at −20 °C. For viral RNA extractions from stool, 140 μl of stool was inoculated with 20 μl of EAV and subjected to an automated extraction on a MagNA Pure 96 nucleic extraction system using the MagNA Pure 96 DNA and the *Viral NA Small Volume Kit* (Roche applied sciences, UK), according to the manufacturers recommendations. PCR amplifications were performed using RNA Master Hydrolysis Probes (Roche applied sciences, UK), and optimized with 1.4 μl of activator on a LightCycler 480II (Roche applied sciences, UK). Thermal cycling was initiated at 61 °C for 3 min for reverse transcription; plates were cooled on ice for 2 min, and amplified for 5 min at 95 °C, followed by 45 cycles at 95 °C for 5 s and 60 °C for 45 s.

### 2.6 Construction of plasmids containing target sequences

PCR amplicons for all target loci were produced using the RT real-time PCR procedure without probe. Amplicons were cloned into pCR 2.1-TOPO (Invitrogen applied biosystems, UK), following the manufacturers guidelines. Plasmid DNA, with cloned target DNA sequences, was purified, and concentrations were calculated using a NanoDrop spectrophotometer (Thermo-Scientific, UK). DNA concentrations were converted to copy number using the formula: mol/g × molecules/mol = molecules/g, via a DNA copy number calculator (http://www.uri.edu/research/gsc/resources/cndna.html). Ten-fold serial dilutions of plasmid DNA containing the cloned target insert were used as an external standard for all quantitative one-step Real-time RT PCR experiments.

### 2.7 Detection limits and amplification interpretation

Ten-fold serial dilutions of plasmid DNA containing the cloned target insert, with concentrations ranging from 5 × 10^0^ to 5 × 10^3^ copies per reaction, were amplified on five consecutive days. Ten replicates were performed daily. The detection limit was calculated using fifty individual *Cp* values and set at the target DNA concentration at which a positive PCR signal was produced in 95% or more of the samples tested.

Accurate DNA extraction and amplification was confirmed by the production of a signal from the EAV internal control. A negative PCR result was concluded if negative controls were negative, if the internal control showed the predicted *Cp* value, and if a reporter signal for *target sequences* could not be detected (*Cp* > 40). Data was deemed non-interpretable when the negative control demonstrated contamination and/or the internal control did not yield a sufficient *Cp* value.

### 2.8 Reproducibility, linearity and efficiency

The co-efficient of variance (CV%) was calculated by assessing the *Cp* value deviation of selected plasmid DNA concentrations across multiple amplifications. Intra-assay reproducibility was determined by comparing the *Cp* values generated in the same run of four replicates over each plasmid concentration. Inter-assay reproducibility was assessed by comparing the *Cp* values generated by four replicates of each plasmid concentration each day over a period of four days. Linearity was assessed from the *Cp* values of 10-fold serial dilutions of plasmid DNA containing cloned target sequences (concentrations 5 × 10^0^ to 5 × 10^8^) and calculating their linear regression. Efficiency was calculated from the slope of the standard curve using the formula: Efficiency = 10^(−1/slope)^−1, according to the methods of Rasmussen (2001).

Data were exported into Microsoft Excel (Microsoft, USA), and analyzed using STATA 9.2 (StataCorp, College Station TX, USA); specific statistical tests used are outlined in the results.

## 3 Results

### 3.1 Analytical specificity and detection limits

Two one-step RT real-time PCR assays were developed. One PCR contained primers and probes detecting RoV, NoVII and EAV, the other contained primers and probes to detect NoVI and EAV. Plasmid DNA containing RoV, NoVI and NoVII target sequences were used as positive amplification controls. Both PCR assays were tested on nucleic acid extracted from 45 gastrointestinal organisms, including enterovirus, *Escherichia coli*, C*ampylobacter*, *Shigella spp*., *Salmonella spp.* and *Klebsiella spp*. Neither assay demonstrated any nonspecific amplification with any target nucleic acid from any of the organisms tested. The NoVI, NoVII and RoV *Cp* values obtained during multiplex PCR remained unchanged from mono-amplification at the same concentration. The detection limits of the assays were 500, 5 and 50 copies of the cloned target sequence for RoV, NoVII and NoVI, respectively.

### 3.2 Reproducibility and linearity

Table 2 shows the results from a series of consecutive standard curve experiments. These data demonstrate overall performance, intra-assay variation and inter-assay variation of the PCRs. The intra-assay CV and the inter-assay CV across the three targets ranged from 0.2% to 4.07% and 0.5% to 5.22%, respectively, with target copy numbers from 5 × 10^0^ to 5 × 10^8^ copies per reaction. Linearity was assessed by *Cp* values generated from amplification of eight ten-fold serial dilutions. The linear regressions of the three standard curves were *R*^2^ = 0.992 for RoV, *R*^2^ = 0.992 for NoVI and *R*^2^ = 0.999 for NoVII, indicating a significant linear correlation between *Cp* value and amount of nucleic acid over a range of concentrations. The efficiencies of the amplifications were 94% for RoV (95% CI; 93–98%), 89% for NoVI (95% CI; 87–91%) and 96% for NovII (95% CI; 94–98%).

### 3.3 Comparison of EIAs and one-step RT real-time PCRs

The performance and sensitivity of the PCR assays were evaluated against EIA (Table 3). Sensitivity was compared using ten-fold dilutions off three RoV EIA+/PCR+ samples and three NoV EIA+/PCR+ samples. For the RoV positive samples, all were PCR+/EIA+ at the 10^−1^ dilution and all were PCR+/EIA− at the 10^−3^ dilution. For the NoVII positive samples, all were PCR+/EIA− at the 10^−3^ dilution. Nucleic acid from RoV PCR+ and NoV PCR+ samples were combined on ten occasions to assess potential amplification inhibition induced by cross-reactivity. The *Cp* values were unchanged in samples containing RoV and NoV from those containing only RoV or NoV.

Ninety-four stool samples from patients with diarrhea and 94 stool samples from patients without diarrhea were investigated by EIA and PCR for RoV and NoV (Table 4). The EIA+ results corresponded precisely with qualitative PCR results for RoV and NoVI/II. In the children with diarrhea, 10/94 (10.6%) samples were PCR+/EIA− for RoV and 22/94 (23.4%) were PCR+/EIA− for NoV. In the children without diarrhea, 0/94 samples were PCR+/EIA− for RoV and 7/94 (7.4%) samples were PCR+/EIA− for NoV. The RoV PCR+/EIA+ *Cp* values samples were compared with the RoV PCR+/EIA− *Cp* values. The median *Cp* value from the RoV PCR+/EIA+ samples was 15.3; the median *Cp* value from the RoV PCR+/EIA− samples was significantly higher at 22.5 (*p* < 0.0001; two-tailed *t*-test). The presence of RoV and NoV in the PCR+/EIA− samples was confirmed by PCR amplification of the VP7 and VP4 genes in the RoV+ samples and VP1 in the NoV+ samples (Yan et al., 2003; Gomara et al., 2001). All produced the expected amplifications. Sequencing of the resulting amplicons confirmed the real-time RT PCR results, demonstrating these samples contained either GI RoV or GII.4 NoV, respectively (data not shown).

### 3.4 Quantitative PCR results

**Fig. 1:**
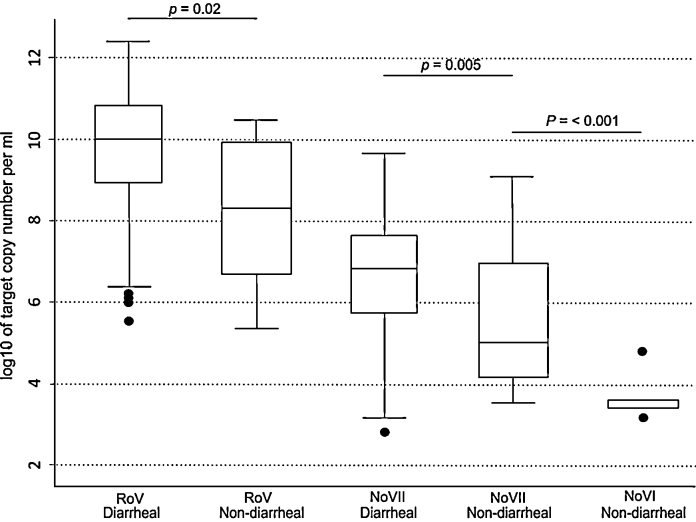
The viral load of RoV, NoVI and NoVII in stool samples. Box plots showing the median and interquartile range of RoV, NoVI and NoVII viral copy number in stool samples from children with and without diarrhea in HCMC, Viet Nam. Statistical significance between the samples from children with and without diarrhea was calculated using a two-tailed *t*-test; significant variation in viral copy number between samples is denoted at the head of the figure; significant *p* values are shown.

The PCRs were used to assess prevalence and quantitate NoV and RoV in stool samples from 217 children with diarrhea and 277 without diarrhea (including the 94 samples used to assess PCR sensitivity against EIA) (Fig. 1). From the samples from children with diarrhea, 101/217 (46.5%) were positive for RoV, 113/217 (52%) were positive for NoVII, 22/217 (10.1%) were positive for both RoV and NoVII and 0/217 were positive for NoVI. From the samples from children without diarrhea, 12/277 (4.3%) were positive for RoV, 25/277 (9%) were positive for NoVII and 5/277 (1.8%) were positive for NoVI.

*Cp* values were converted to copy number by use of a standard curve. The RoV loads in the samples from children with diarrhea were significantly higher than in the samples from children without diarrhea (with diarrhea; median = 10.06 log/RNA copies/ml (range; 5.56–12.49), without diarrhea; median = 8.33 log/RNA copies/ml (range; 5.43–10.52), *p* < 0.001, two tailed *t*-test) (Fig. 1). NoVII loads in the samples from children with diarrhea were significantly higher than in the samples from children without diarrhea (with diarrhea; median = 6.85 log/RNA copies/ml (range; 2.89–9.71), without diarrhea; median = 5.07 log/RNA copies/ml (range; 3.63–9.16), *p* = 0.02, two tailed *t*-test). Only samples from children without diarrhea tested positive for NoVI, with the viral load (log/RNA copies/ml) ranging from 3.2 to 4.88; median 3.56.

## 4 Discussion

Here we have developed, validated and used a real-time RT-PCR assay to detect and quantify the two major causes of viral diarrhea. The performance of the validated assays was controlled by the inclusion of a standardized internal control. The internal control addition permits assurance of extraction, amplification, and quantitation, making these assays a robust for use in a clinical microbiology laboratory. Furthermore, these assays were tested throughout a large range of nucleic acid concentrations, producing reliable and reproducible quantitative results.

The target regions for the primers and probes for NoV and RoV have been well characterized and were originally designed by Pang et al. (2004) and Kageyama et al. (2003) for RoV and NoV, respectively. These methods have evolved and the primers and probes have been modified, incorporating degenerate nucleotides to account for known diversity within the target regions (Freeman et al., 2008; Trujillo et al., 2006). However, the true specificity and sensitivity of the modified primers and probes have never been directly assessed. The optimized conditions of the virus specific PCRs presented above demonstrated an increase in sensitivity over the current universal gold standard, EIA. The PCR method consistently detected NoV and RoV in lower concentrations than EIA. However, corroborating this increase in sensitivity over the gold standard is a challenge, as nonspecific hybridization at low DNA concentrations may induce a comparable result. Yet, the proportion of PCR+/EIA− samples was greater in the samples from children with diarrhea than the samples from children without diarrhea and conventional PCR and amplicon sequencing confirmed the real-time RT PCR data. Furthermore, the PCR+/EIA− samples exhibited significantly higher *Cp* values than the PCR+/EIA+ samples, corresponding with the stool dilution experiments. Taken together, these data strongly suggest that the PCR assays are amplifying nucleic acid from RoV and NoV exclusively, rather than demonstrating cross-hybridization.

A noteworthy observation from the development of these assays was the ability to detect NoV and RoV in the stools of individuals with diarrhea. NoV and RoV infections without diarrheal symptoms have been described previously (O’Ryan et al., 2009; Walther et al., 1983; Zhang et al., 2011), and due to the design of the study presented above, it is impossible to assess the role of these individuals in Ho Chi Minh City. However, NoV and RoV the stools of individuals without diarrhea may reflect a true asymptomatic infection or longer-term shedding after a symptomatic infection (Eiden et al., 1988; Phillips et al., 2009). Furthermore, as the incubation period for NoV and RoV is approximately two days (Bernstein, 2009; Glass, Parashar, and Estes, 2009), and both pathogens have an exceptionally low infective dose (Glass et al., 2009) (Leung, Kellner, and Davies, 2005; Leung and Pai, 1988), these PCR+ individuals may be in the incubation period. The PCR presented above can be used to address this phenomenon in a prospective study.

Collectively, RoV and NoV are the leading causes of acute viral diarrhea and are a substantial cause of mortality in children under five years old worldwide. Co-infection with both pathogens is common and assays have been developed for the detection of multiple causes of viral gastroenteritis, including NoV, RoV, astrovirus and adenovirus (Feeney et al., 2011; van Maarseveen et al., 2010). The work presented above augments the PCR methods of Feeney et al. (2011) and van Maarseveen et al. (2010), describing an increase in sensitive and offering a reliable solution or alternative to the diagnosis of these pathogens. The addition of an internal control means that these methods are transferable, and can be used for diagnosis, environmental sampling and epidemiological screening. Furthermore, the ability to perform quantitation permits a greater degree of utility, facilitating an understanding of the dynamics of viruses prior to, during and post-infection. In conclusion, the work presented above outlines an internally controlled multiplex real-time RT PCR assay to detect RoV and NoV in stool samples. The methodology is robust, exhibits a high degree of reproducibility and may have a greater utility and sensitivity that commercial EIA kits.

## Competing interests

The authors wish to declare they have no competing interests.

## Figures and Tables

**Table 1:** Sequences and concentrations of primers and probes used in this study.

Viral target	Target region/amplicon size	Primer/probe name	Sequences^a^	Final conc. (μM)	Reference
RoV	Non-structural protein 3 (NSP3)/87 bp	NVP3-FDeg	ACC ATC TWC ACR TRA CCC TC	1	
		NVP3-R1	GGT CAC ATA ACG CCC CTA TA	1	Freeman et al. (2008)
		NVP3-Probe	FAM-ATG AGC ACA ATA GTT AAA AGC TAA CAC TGT CAA-BHQ1	0.125	

NoVII	ORF1-ORF2 junction/98 bp	Cog 2F	CAR GAR BCN ATG TTY AGR TGG ATG AG	1	
		Cog 2R	TCG ACG CCA TCT TCA TTC ACA	1	Trujillo et al. (2006)
		Ring 2	Cyan500–TGG GAG GGC GAT CGC AAT CT–BHQ1	0.125	

NoVI	ORF1-ORF2 junction/84 bp	Cog 1F	CGY TGG ATG CGI TTY CAT GA	1	
		Cog 1R	CTT AGA CGC CAT CAT CAT TYA C	1	Trujillo et al. (2006)
		Ring 1 C	FAM–AGA TYG CGI TCI CCT GTC CA–BHQ1	0.125	

EAV		EAV-F	CAT CTC TTG CTT TGC TCC TTA G	0.2	
		EAV-R	AGC CGC ACC TTC ACA TTG	0.2	Hue et al. (2011)
		EAV-probe	Cyan500–CGC GCT CGC TGT CAG AAC AAC ATT ATT GCC CAC AGC GCG–BHQ3	0.05	

a W = T, U, A; R = A, G; Y = C, T; B = C, G, T; N = any; I = inosine.

**Table 2:** Assessment of the reproducibility (CV%) of the assays on diluted plasmid DNA containing cloned target sequences.

Target and variable	Target concentration								
	5 × 10^8^	5 × 10^7^	5 × 10^6^	5 × 10^5^	5 × 10^4^	5 × 10^3^	5 × 10^2^	5 × 10^1^	5 × 10^0^
RoV									
Intra-assay variation^a^	4.07	0.2	0.77	1.87	1.43	1.12	1.14	–	–
Inter-assay variation^b^	4.91	5.22	1.83	2.77	0.98	0.66	1.43	–	–

NovI									
Intra-assay variation	2.06	0.3	0.81	0.55	0.94	0.3	0.95	1.96	–
Inter-assay variation	1.73	3.28	2.01	0.5	0.79	1.68	2	1.64	–

NoVII									
Intra-assay variation	0.51	0.71	0.58	0.58	0.27	0.23	0.41	0.36	1.02
Inter-assay variation	3.42	1.13	1.24	0.9	3.36	2.77	2.54	2.56	3.02

a Intra-assay variation was calculated by measuring the co-efficient of variance of the *Cp* value on four concurrently run assays.

b Inter-assay variation was calculated by comparing variation in *Cp* value on four independently run assays.

**Table 3:** Analytical comparison of the performance of real-time PCR against EIA.

Viral sample	Test	Concentration of diluted RNA				
		Neat	10^−1^	10^−2^	10^−3^	10^−4^
RoV samples (sample ID)						
30,217	RT-PCR	15.32	18.08	21.39	24.68	27.89
	EIA	Positive	Positive	Positive	Negative	Negative
30,205	RT-PCR	17.8	19.48	22.68	26.29	29.52
	EIA	Positive	Positive	Positive	Negative	Negative
30,453	RT-PCR	17.73	20.6	23.65	26.9	29.43
	EIA	Positive	Positive	Negative	Negative	Negative

NoV samples (sample ID)						
20,154	RT-PCR	15.35	17.7	21.54	25.43	30.02
	EIA	Positive	Positive	Positive	Negative	Negative
20,614	RT-PCR	17.52	20.22	24.23	27.4	31.4
	EIA	Positive	Negative	Negative	Negative	Negative
20,172	RT-PCR	18.31	21.14	24.76	28.18	32.8
	EIA	Positive	Negative	Negative	Negative	Negative

**Table 4:** Performance of RT Real-time PCR in comparison to EIA.

Assay result		Diarrheal group (*n* = 94)		Non-diarrheal group (*n* = 94)	
Real-time PCR	EIA	RoV	NoV	RoV	NoV
−	−	50	47	94	86
+	+	34	25	0	1
−	+	0	0	0	0
+	−	10	22	0	7
Increase in sensitivity		10.6% (10/94)	23.4% (22/94)	0% (0/94)	7.4% (7/94)
